# Multi-Criteria Analysis for the Prioritization of Areas for the In Situ Conservation of *Crataegus* L., an Underutilized Fruit Tree in Mexico

**DOI:** 10.3390/plants10122561

**Published:** 2021-11-23

**Authors:** Karina Sandibel Vera-Sánchez, Mauricio Parra-Quijano, Raúl Nieto-Ángel, Alejandro F. Barrientos-Priego

**Affiliations:** 1 Posgrado en Horticultura, Departamento de Fitotecnia, Universidad Autonoma Chapingo, Texcoco 56230, Mexico; r.nietoangel@gmail.com (R.N.-Á.); abarrien@gmail.com (A.F.B.-P.); 2 Facultad de Ciencias Agrarias, Universidad Nacional de Colombia Sede Bogotá, Bogotá 14490, Colombia; hmparraq@unal.edu.co

**Keywords:** tejocote, in situ conservation, Mexican hawthorn, underutilized crops, agrobiodiversity

## Abstract

Complementary ex situ and in situ conservation, including the on-farm alternative, is a highly desired and dynamic strategy that allows the natural evolution of the conserved germplasm. Due to the high costs involved, in addition to the limitations of both economic and human resources, in situ conservation must focus on areas where the greatest benefits are obtained, and the efforts made result in better impacts. Therefore, using spatial multi-criteria analysis and expert knowledge, 22 and 23 criteria were obtained as important for the conservation of wild and cultivated hawthorn, respectively. Criteria weights were calculated by the analytic hierarchy process and expert knowledge. The results showed species richness, phenotypic and ecogeographic diversity, and areas not covered by the official protected areas network were the most important criteria for in situ conservation of wild hawthorn. Prioritized areas were particularly focused in Chiapas, State of Mexico and Morelos. The prioritized areas for the in situ conservation of cultivated hawthorn were mostly defined by criteria such as number of cultivated varieties, number of uses, phenotypic diversity, ecogeographical diversity, and areas with rainfed agriculture. These areas were located mainly in Puebla. From this study, we propose a list of priority areas for the in situ conservation of both cultivated and wild hawthorn.

## 1. Introduction

Biodiversity has been highlighted as contributing to the productivity, sustainability, and stability of agricultural systems [1,2]. The accelerated loss of biodiversity has caused growing concern, and even a significant extinction of species has been reported due to the consequences of climate change and land use [3]. For this reason, the design of strategies and programs for the knowledge, conservation, management, and sustainable use of biodiversity is important [4,5,6,7]. Cultivated plants have been mainly preserved through ex situ conservation in germplasm banks, whereas in situ conservation has been carried out in growers’ farms to a lesser extent, and wild species have been mostly conserved in nature reserves [3,8]. In situ and ex situ conservation strategies should not be exclusive of each other, but complementary [9]. Due to the high costs of the conservation of large areas and the limitations of economic and human resources, in situ conservation must focus on zones where the greatest benefits are obtained and the efforts made result in greater impacts [10,11]. For this conservation to be effective, it is important to know and analyze the characteristics of the places where action must be taken to obtain the best results. A valuable tool to guide and optimize conservation is the identification of priority areas [6,11,12,13]; this identification must be carried out under a structured, scientifically defensible, and effective framework for the protection of biodiversity [14]. Spatial planning projects and the allocation of conservation zones must consider social, economic, cultural, ecological, geographical, and biological aspects (biodiversity, species subsets, habitat types, and environmental classes) [15,16,17].

In Mexico, different approaches and methodologies have been developed for the identification of priority areas for the conservation of biological diversity. These methodologies range from optimization methods based on expert knowledge and participatory approaches to spatial statistical models [11,12,18,19]. In situ conservation actions have been specifically implemented in the establishment of protected natural areas [20] or terrestrial priority areas for biodiversity conservation [21]. Additionally, these actions have been carried out to protect agricultural biodiversity as in the case of maize [22] and priority sites for the in situ conservation of crop wild relatives (CWR) [23].

Due to the complexity of the natural environment caused by the intervention of multiple interactive variables and their response to human action, multi-criteria analysis (MCA) has been used as a useful decision-making tool [16,24]. MCA allows the selection, analysis, and combination of several criteria and indicators to generate hierarchical solutions [25]. One modality of MCA is the incorporation of the knowledge of experts, assigning weights to each criterion and indicating the relative importance between them [26]. MCA techniques have been used in multiple studies on biodiversity and the definition of suitable areas [16,27,28] because they can combine agronomic, ecological, sociocultural, and economic information [17]. The combination of MCA, spatial analysis, and map algebra represents a complete, efficient, and economic methodology to be applied at regional and local scales for the identification of priority areas for in situ conservation [11]. Furthermore, the conjunction between geographic information systems (GIS) and MCA has been highly productive in the management and resolution of environmental and territorial problems [24]. MCA has been commonly used for the conservation of marine or animal biodiversity [29,30,31,32]. In some studies, it has been applied to multi-species cases where several plant species have been involved [33,34,35]. However, there is no report whatsoever of the use of MCA in the case of agrobiodiversity, only for the determination of priority conservation areas of maize in the Sierra of Ecuador [35]. Nevertheless, it has been used in Mexico for prioritizations of areas for the conservation of terrestrial biodiversity [6,12,13,21,22,23], restoration of ecosystems [10,11,12,36,37], and even for the collection of germplasm [38].

The genus *Crataegus* L. is distributed in temperate regions of the world, consisting of 140 to 200 species [39]. Approximately 15 species have been reported in Mexico [40,41], where they are commonly known as “tejocote” which means “hard fruit” in Nahuatl [42]. Tejocote, also known as “hawthorn” in English, represents an important economic and cultural value, as it is consumed as fresh fruit, in drinks, and in handcrafted sweets. It has medicinal properties and nutritional and ecological relevance because it can be used as rootstock and it is a source of high content of Vitamin A, Vitamin C, minerals, oligomeric procianidines, triterpenes, carotenes, flavonoids, polysaccharides, and catecholamines [43,44]. Globally, the species of *Crataegus* L. have been utilized since ancient times to treat health disorders due to their cytotoxic, gastroprotective, anti-inflammatory, anti-HIV, antimicrobial, and antioxidant biological activity [45]. In some Mexican localities, tejocote is used for treating flu and coughing, and also to prevent several cardiovascular diseases [46]. The leaves and flowers in infusion are used for kidney diseases, and also to lose weight, because it is an excellent diuretic and cleans urinary tracks. It also moderates contractions in the case of tachicardy and is used as an anti-spasm treatment, due to the excellent anti-spasm properties of the root and tree bark [44,47]. Tejocote fruit has been traditionally utilized mainly as an essential decorative element on altars in the “Day of the Dead” celebration, and as one of the most important ingredients of the traditional “ponche”, a hot fruit punch beverage specially prepared at this time. In the traditional parties called “posadas” that take place before Christmas, tejocote fresh fruit are found inside “piñatas”, which are decorated boxes containing fruits, candies, and little gifts. In addition, due to their morphological characteristics they are suitable for use as ornamental plants, although the concept of the ornamental tejocote tree in urban landscape design has not been fully explored [46]. Currently, the products derived from tejocote are sold mainly as preserved fruit, jelly, candy, extract, wine and in capsules, derived from the leaves, flowers and fruits. However, the economic potential of the plant is increasing and new presentations of food products made with a hawthorn base have been proposed that preserve its biological and antioxidant properties [44].

Hawthorns are mainly distributed as wild species in the mountainous areas of Mexico [43]. Despite being considered an underutilized fruit tree [48], commercial crops (mainly of *C. mexicana*) have been documented in six states, highlighting Puebla (95% of the production) with more than 900 ha harvested, production of five thousand tons, and earning almost USD 1 million for the year 2018 [49]. Although the USA border was opened to Mexican hawthorn in 2015, there are no statistics about exportation.

Five hawthorn varieties of common use have been registered as an initiative for the legal protection of its diversity in Mexico [50], which have been introduced in crops because they show desirable characteristics for growers. Currently, approximately 160 accessions of 11 species are kept at the Mexican hawthorn germplasm bank of the Universidad Autonoma Chapingo, as an ex situ conservation strategy. This conservation alternative is important because the area with new hawthorn crops and its commercial activity are increasing, which may reduce its diversity [51]. In addition, ten species of Mexican hawthorn were previously assessed for the global Red List [52], of which *C. aurescens*, *C. cuprina*, *C. grandifolia*, and *C. johnstonii* are endangered, thus driving the design of strategies for their conservation.

Thus, the objective of this study was to identify priority areas for the in situ conservation of hawthorn (*Crataegus* spp.) diversity through the MCA methodology, as a useful tool to guide and optimize efforts in project design, and public and private programs on the conservation of hawthorn biodiversity. The assumption of this study is that the sites for in situ conservation selected by multi-criteria analysis can improve decision-making processes about where to invest in projects or programs to promote the maximum long-term conservation of Mexican hawthorn. This would not be achievable by either selecting the sites randomly or selecting from the perspective of a reduced group of decision makers or experts.

## 2. Results and Discussion

Seventeen responses were obtained from the online survey—twelve corresponded to academics, two to government authorities, two to growers, and one to a supplier of hawthorn-based products. The number of experts in this study was two less than the number of experts gathered (19) to carry out the proposal of ecosystem restoration priorities in Mexico [36].

### 2.1. Important Criteria for the In Situ Conservation of Hawthorn

From the information of the surveys, 22 important criteria (80% of the total proposed) were selected for wild hawthorn. These criteria obtained more than 50% of evaluations as “important criteria” (Table 1). The three most important criteria for the experts were of the biological type (species richness, and phenotypic and ecogeographic diversity), which were the same obtained by the AHP pathway. This is consistent with two studies that indicate that priority areas for conservation should not only be represented by the groups of species but also by the environmental diversity to capture the species diversity that occurs correlated with specific environmental characteristics [53,54]. Species richness is also highlighted as one of the most used criteria in systematic conservation planning because it is one of the indicators that is easy to estimate and interpret [23,55,56]. However, this parameter alone does not appropriately indicate conservation priorities [18]. Conversely, the criteria with the lowest weights assigned by the experts were areas with submontane grasslands and shrublands, population density, and percentage of the population employed in agricultural activity. For AHP, the criteria with the lowest weights were the number of common names and harvest of wild hawthorn for sale (Table 1).

For cultivated hawthorn, 23 criteria were selected (70% of the total proposed) because they obtained more than 50% of evaluations as “important criteria” (Table 2). For the experts, the three most important criteria were the number of cultivated varieties, number of uses, and phenotypic diversity. In this regard, another study states that varietal richness in conjunction with other indicators such as spatial evenness and between- and within-variety genetic diversity can give an approximation of crop diversity and may be of particular interest for the elaboration of instruments focused on the prevention of genetic erosion [57].

The criteria with the lowest weights were sites with high priority for biodiversity conservation, number of uses, and percentage of the population living in poverty. The first criterion may indicate sites of high wild biodiversity and their level of risk [37], which are probably not directly related to *C. mexicana* as a cultivated species. Regarding the poverty criterion, two contrasting ideas were discussed. The first mentions that poverty can be eradicated with biodiversity conservation programs. The second indicates that these programs may fail in areas with a high poverty rate [58]. Poverty may cause environmental degradation in developing countries such as Mexico. A very important aspect to prevent the overexploitation of natural resources is relieving poverty, because poor people are most dependent on the exploitation of biodiversity. Giving these people access to economic resources, knowledge, and opportunities of development is the most efficient way to avoid overexploitation of natural resources [59]. Because *C. mexicana* is a cultivated species, the economic level may not be a relevant factor for its conservation.

According to the weights assigned by AHP for cultivated hawthorn, the most important criteria were species richness, ecogeographic diversity, and rainfed agriculture areas. The first is the criterion with the greatest weight; however, it may represent a bias because AHP paired comparisons were carried out with only two experts. This can be corrected in subsequent studies if paired comparisons are carried out between at least three experts. The least important criteria were the estimated age of the established plants, hawthorn cultivation for sale, and common names of cultivated hawthorn, all criteria related to cultural aspects.

### 2.2. Highly Important Criteria for the In Situ Conservation of Hawthorn

For both HIC-EXP and HIC-AHP, 11 highly important criteria were determined for the in situ conservation of wild hawthorn (Table 1), adding an importance of 61.35 and 81.06 (obtained from de sum of HIC and multiplied by 100), respectively. Thirty-six percent of HIC-EXP are cultural criteria, which include the number of different types of wild hawthorn, loss of different wild hawthorn ecotypes, loss of different wild hawthorn ecotypes specifically caused by the lack of knowledge about their uses, lack of consumption and/or lack of cultivation, and harvest of wild hawthorn for self-consumption, followed by biological and ecological criteria (both with 27%). The opposite was observed for HIC-AHP where the least important criteria are the cultural (18%), whereas biological, ecological, and socio-economic criteria are equitably represented (27%). Both HICs agree in 55% of the criteria, with the following in common: species richness, phenotypic diversity, ecogeographic diversity, number of different types of wild hawthorn, loss of different types of wild hawthorn, and protected natural areas. This indicates that there is some level of biodiversity protection in PNAs. However, it is highlighted that many of the protected areas were chosen for their scenic beauty or opportunistically, without an evaluation that would direct priorities or ensure a priori an adequate representation of biodiversity, especially of the most vulnerable or at risk [18]. It is important to mention that unlike in the HIC-AHP, in HIC-EXP criteria, such as the level of indigenous presence, type of municipality, and areas with temperate forests or submontane grasslands and shrublands, were discarded, although the last two criteria refer to the vegetation related to the climates where the species of the genus *Crataegus* L. are mainly distributed [34,40]. The exclusion of the criterion related to the level of indigenous presence is also in contrast with studies [60] that state that indigenous populations are an essential component of conservation and ecotourism programs. In addition to preserving the depositories of an enormous biological, landscape, and natural diversity, these programs also allow indigenous people the reappropriation and reuse of basic natural resources for their existence.

For cultivated hawthorn, 12 highly important criteria were defined for HIC-EXP and HIC-AHP, with accumulated importance of 62.79 and 82.9, respectively (Table 2). The sets correspond to 63.6% of the criteria, among which the number and type of cultivated varieties, phenotypic and ecogeographic diversity, and type of municipality stand out. In HIC-EXP, the intervention of cultural criteria is evidenced because they represent 75%, whereas those excluded are of the ecological type. The number of uses, substitution by new varieties, and seed flow stand out among the exclusive criteria of this set. Seed flow is regarded as a dynamic process in which variability is selected and introduced through the free exchange of materials between communities. One of its advantages is the adaptation to marginal environments and biotic and abiotic stresses, with conservation linked to seed use and evolutionary process [3]. In HIC-AHP the socio-economic and cultural criteria (both with 30%) are those that are mostly represented. The exclusive criteria in this set are species richness, percentage of the population employed in agricultural activity, percentage of elderly people, percentage of the population living in poverty, and rainfed agriculture areas.

### 2.3. Maps of Priority Areas for the In Situ Conservation of Hawthorn

**Figure 1:**
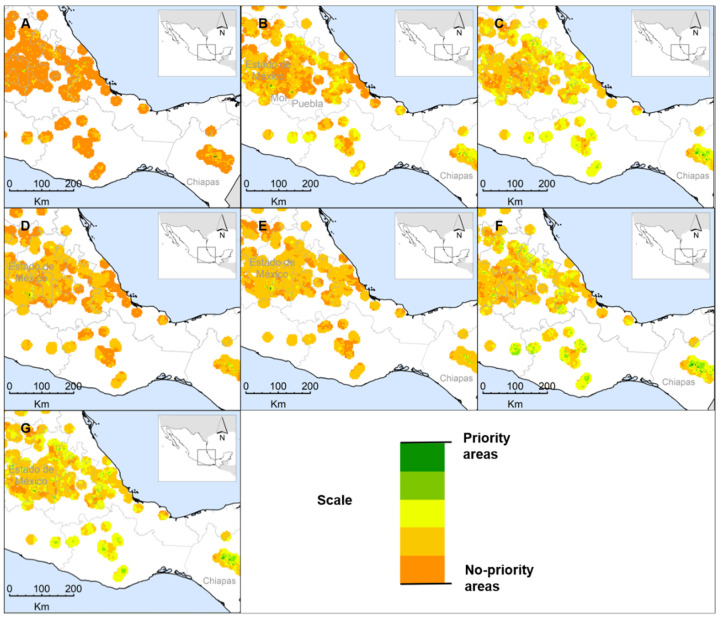
Priority area scenarios for the in situ conservation of wild hawthorn (*Crataegus* spp.), based on seven different criteria-map combinations. (**A**) S_1_—All criteria with the same weight. (**B**) S_2_—All criteria with weights assigned by experts. (**C**) S_3_—All criteria with weights assigned by AHP. (**D**) E_4_—HIC-EXP with the same weight. (**E**) S_5_—HIC-EXP with weights assigned by experts. (**F**) S_6_—HIC-AHP with the same weight. (**G**) S_7_—HIC-AHP with weights assigned by AHP.

The summarized criteria map resulted in seven different priority area scenarios. For wild hawthorn, scenarios S_1_ and S_6_ show differences in the distribution pattern of these areas but reduce their specific location to Chiapas. The first scenario shows only two high-priority cells in San Cristobal de las Casas and Huixtan, whereas the second scatters seven cells in five municipalities, becoming the scenario that covers the largest surface of priority areas (175 km^2^). These results are more specific and match a study that indicated that the mountainous region of Chiapas corresponded to one of the priority areas for the conservation of the genus *Crataegus* L. diversity [38]. Scenarios S_4_ and S_5_ have practically the same distribution pattern; both consider 50 km^2^ and locate the priority areas in three municipalities of the State of Mexico (Xalatlaco, Tianguistenco, and Ocuilan). This suggests that HIC-EXP with the same weights and HIC-EXP with relative weights by experts provide similar information in the definition of priority areas. S_7_ is the scenario with the second-largest area (125 km^2^) and locates the priority areas in the State of Mexico and Chiapas. Finally, S_2_ is the only scenario that concentrates the priority areas in three states, Chiapas, Morelos, and the State of Mexico (Figure 1). Although all the cells with high priority are in areas considered to be priorities for the collection and conservation of *Crataegus* spp. diversity [43], this location probably also reflects a slight bias due to the information provided by the surveys and the phenotypic diversity of the characterized accessions, because this criterion-map was obtained from the information available for 140 accessions conserved in the germplasm bank [51].

**Figure 2:**
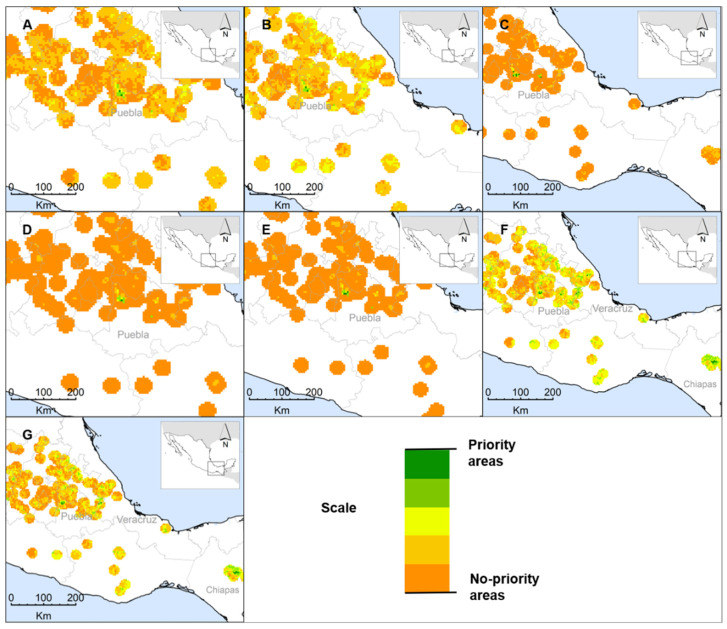
Priority area scenarios for the in situ conservation of cultivated hawthorn (*C. mexicana*), based on seven different criteria-map combinations. (**A**) S_1_—All criteria with the same weight. (**B**) S_2_—All criteria with weights assigned by experts. (**C**) S_3_—All criteria with weights assigned by AHP. (**D**) S_4_—HIC-EXP with the same weight. (**E**) S_5_—HIC-EXP with weights assigned by experts. (**F**) E_6_—HIC-AHP with the same weight. (**G**) S_7_—HIC-AHP with weights assigned by AHP.

The priority area scenarios for *C. mexicana* show that the first five (S_1_, S_2_, S_3_, S_4_, S_5_) concentrate the cells with priority only in Puebla (Figure 2 and Table 3). Of these scenarios, S_1_ and S_2_ maintain a similar distribution pattern and even the priority areas are the same. S_3_ distributes six priority cells in 13 municipalities. S_4_ represents the smallest area (25 km^2^) in Calpan, Nealtican, and San Nicolas de Los Ranchos. S_6_ locates the priority areas in Puebla and Chiapas, with a surface of 375 km^2^; this is the largest of all the scenarios, even when compared to the wild ones. Finally, S_7_ concentrates the priority areas in Puebla, Chiapas, and Veracruz with a surface of 325 km^2^, although hawthorn has not been documented as being cultivated in Chiapas and Veracruz. Puebla is the only state where hawthorn cultivation has been recorded and the presence of plantations has been verified, specifically in the municipalities of Soltepec, Chiautzingo, and Calpan, which represent more than 50% of the surface reported for this state [49]. In Puebla, in situ conservation actions have been carried out through participatory plant breeding with growers to select materials with outstanding yield and fruit quality [61]. These actions are promoted in the World Action Plan for the in situ conservation of Plant Genetic Resources for Food and Agriculture (PGRFA) [62].

It is important to highlight that AHP criteria sets are included in scenarios S_3_, S_5_, and S_6_, where “species richness” was the criterion with the greatest importance. This fact may not only explain that these scenarios increase their surface area, but also that the priority zones are extended to other places where species richness is probably high, such as Chiapas and Veracruz. However, in these sites, a valuable genetic source of *C. mexicana* can be found for plant breeding programs, which can be useful in the development of hawthorn varieties with beneficial characteristics to growers. As some studies suggest, wild species related to crops and collection species are also worthy of conservation actions, because they correspond to an important set of genetic resources due to their potential for use [63,64,65,66].

An important recommendation for using expert knowledge is to consider a high number of experts from different sectors due to the “wisdom of the crowd”, which suggests that the larger the group of experts, the more likely it is to find the optimal solution [67]. In addition, online surveys make it easier to reach experts from all over the world and the time spent answering them is reduced to minutes. By comparison, AHP paired comparisons take hours and specialists usually do not have available time to carry them out.

### 2.4. Recommendations for Decision-Makers

Based on highly important criteria, it can be recommended to decision-makers on agrobiodiversity conservation in Mexico that new priority areas for the in situ conservation of wild hawthorn (*Crataegus* spp.) should be in zones with high species richness, high phenotypic and ecogeographic diversity, in rural municipalities with a high percentage of the population living in poverty, and with the presence of indigenous populations. Additionally, these priority areas must be associated with vegetation such as temperate forests or submontane grasslands and shrublands, preferably outside protected natural areas or with a low level of conservation, where a larger number of wild hawthorns are harvested mainly for self-consumption and the threat of loss of these hawthorns has been registered. 

The priority areas for the conservation of cultivated hawthorn (*C. mexicana*) should be in sites where many mostly traditional hawthorn varieties are grown. Additionally, there should be no loss of these varieties and they should be associated with a high number of different crops. These areas should also be in rural municipalities, specifically in zones with rainfed agriculture where a high percentage of the population is dedicated to agricultural activities and lives in poverty. The zones should preferably be in areas with high phenotypic and ecogeographic diversity.

Although the different scenarios offer similar options for priority areas, each one displays unique cells. Therefore, the selection of the strategy to determine priority areas for the implementation of programs or projects for the in situ conservation of one or several target species (in this case *Crataegus* spp.) will depend on the decision-makers and the available economic resources.

## 3. Materials and Methods

### 3.1. Species and Study Area

**Figure 3:**
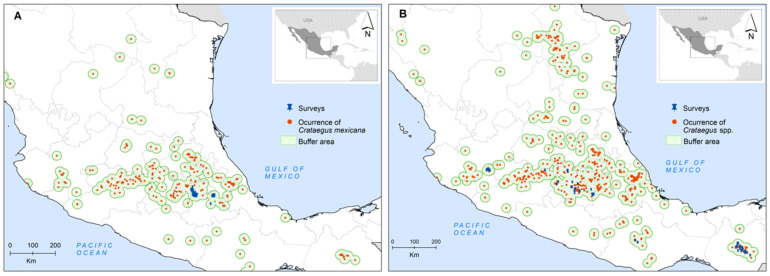
Geographic distribution, survey sites and working area for (**A**) cultivated and (**B**) wild hawthorn.

A spatial multi-criteria analysis (sMCA) was carried out for (1) *C. mexicana* as a cultivated species, documented in crops in Mexico, specifically in Puebla (hereinafter referred to as “cultivated”), and (2) other species of the genus *Crataegus* L., except *C. mexicana*, that grow under natural conditions and are whose fruits are harvested (hereinafter referred to as “wild”). The study area was defined based on the occurrence data from herbaria, Global Biodiversity Information Facility (GBIF) records [68], and collection sites with a buffer area of 20 km from each point of occurrence. The resulting working area was 10,955 cells with a resolution of 2.5 arc-min (5 km × 5 km at the equator) in the case of wild species, whereas for cultivated species it was 6007 cells. These cells represent 13% and 8% of the national territory, respectively (Figure 3).

### 3.2. GIS Building of Criteria

A list of criteria to be considered in the definition of areas for in situ conservation was drawn up with the support of a network of researchers working on *Crataegus* spp. in Mexico [61]. Twenty-eight and thirty-four criteria were collected for *C. mexicana* and *Crataegus* spp., respectively. The criteria were classified as biological, socioeconomic, ecological, and cultural (Table S1). The information on the cultural criteria was obtained from surveys applied to 56 growers and 48 collectors of hawthorn in 2016 (Figure 3). For the rest of the criteria, information was collected from different sources [20,21,69,70,71,72,73,74,75,76,77,78]. In a geographical information system (GIS), criterion-maps were created and masked for the working area, all in raster format, and representing the georeferenced information of the sources (Table S1). Thus, the set of maps was standardized, all having the same geographic coverage, resolution, and coordinate system (WGS-84) in such a way that they could be spatially superimposed and be ready for mathematical operations.

### 3.3. Selection of Criteria and Reclassification of Scales

To select important criteria and how they would influence the definition of priority areas, an online survey was carried out with experts in the study, conservation, management, and use of *Crataegus* spp. genetic resources, fruit trees, and agrobiodiversity in general. The group of experts included academics, growers, and government authorities who are involved in research, conservation, and utilization of Mexican hawthorn or are coordinating actions for the conservation of biodiversity and plant genetic resources. Experts were asked to decide whether each criterion was important or not for the in situ conservation of *Crataegus* spp. If the answer to the criterion’s importance was negative, the survey moved to the next criterion. If the answer was positive, the experts were asked to rate the criterion’s importance on a scale from 1 to 10, with 1 being the least and 10 the most important. Finally, they were asked to indicate the status that would be favorable for conservation for each criterion they considered important. For example, for the criterion “Species richness” there were two statuses, high or low richness; then, the expert had to indicate which of the two was the most appropriate for in situ conservation according to their knowledge and experience. The query forms can be viewed at the following links: wild: http://goo.gl/VdRFWB (accessed on 01 September 2021 and cultivated: https://goo.gl/CDfp7y (accessed on 01 September 2021).

Subsequently, an initial filter of criteria was carried out; those criteria that obtained more than 50% of responses as “not important criterion” were eliminated, in addition to those that had a tie between the two possible statuses for conservation (favorable and unfavorable).

The criterion-maps valued as important were reclassified based on the information from the experts on the beneficial status for conservation, using a scale from 1 to 100, where the favorable status was assigned high values (close to or equal to 100). The original source was used for this transformation, in which each criterion status (qualitative or quantitative) was assigned a numerical and increasing value depending on how favorable it was for conservation. For example, the qualitative criterion “indigenous presence” originally had four statuses (no population, low, medium, and high). Then, each status was assigned a value on the scale from 0 to 100, where 0 corresponds to “no population”, 25 to “low”, 75 to “medium” and 100 to “high”, following expert knowledge that pointed out that a high level of indigenous population is favorable for the in situ conservation of hawthorn. The quantitative criterion “percentage of the population living in poverty” showed values from 8 to 97 [70]; in this case, percentages were grouped into five quantile intervals. The values from “8 to 51” corresponded to 20, “52 to 64” to 40, “65 to 76” to 60, “77 to 85” to 80, and “86 to 97” to 100. For bimodal criteria (with two possible responses or statuses), reclassification was limited to 0 and 100 or vice versa, depending on how favorable each status was for each criterion according to expert knowledge.

### 3.4. Weight Calculation and High Importance Criteria Sets

Weight calculation was carried out in two ways: (1) The Expert System (EXP), using the importance that the experts assigned to each criterion on a scale from 1 to 10. For this, once the survey ratings were consolidated, a normalized matrix was constructed (dividing each score by the sum of scores for each criterion). The weighted average was obtained and finally, the weight for each criterion was calculated. (2) The analytic hierarchy process (AHP) based on paired comparisons of the criteria to structure a decision-making process in a scenario affected by multiple factors [79]. Researchers working on *Crataegus* spp. [61] carried out the paired comparisons using the Saaty scale [80]. The matrix obtained was normalized and weights were calculated for each criterion.

Two sets of criteria were determined by calculating the 50th percentile of relative weights. The criteria assessed by experts that were above this threshold formed the set of highly important criteria (HIC-EXP). Under the same procedure, but using relative weights based on AHP, the second set of highly important criteria (HIC-AHP) was generated.

### 3.5. Sum of Criteria and Maps of Priority Areas

**Figure 4:**
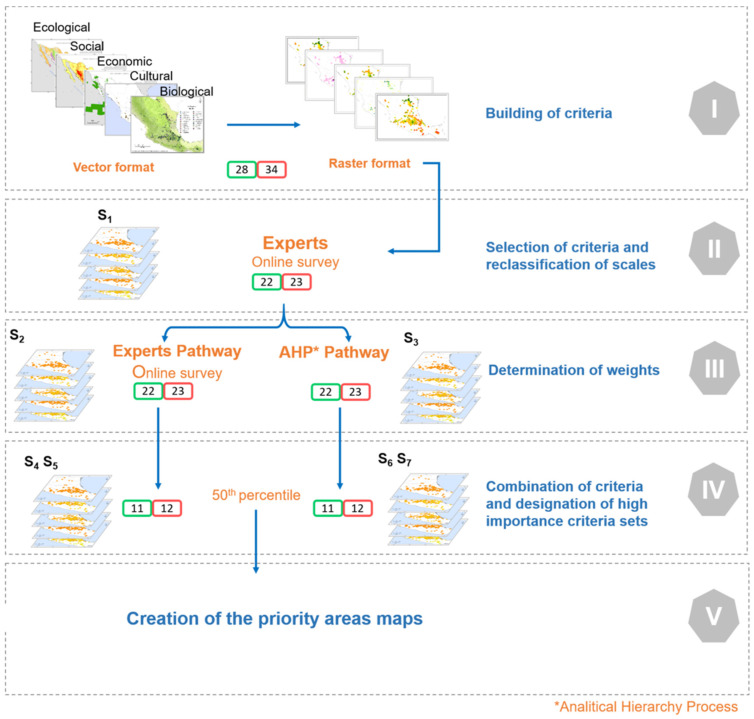
Process for the identification of priority areas for the in situ conservation of wild hawthorn (*Crataegus* spp.) and cultivated hawthorn (*C. mexicana*) in Mexico. The green and red boxes indicate the number of criteria considered for both wild and cultivated hawthorn at each stage of the process, respectively. S_1_, S_2_, S_3_, S_4_, S_5_, S_6_, and S_7_ represent the comparison scenarios.

To compare different scenarios to identify priority areas, seven prioritization map-response combinations were defined: 1. All criteria with the same weight (S_1_), where all criteria are equally important (simple sum). 2. Criteria with weights assigned by experts (S_2_). 3. Criteria with weights assigned by AHP (S_3_). 4. HIC-EXP with the same weight (S_4_). 5. HIC-EXP with their weights (S_5_). 6. HIC-AHP with the same weight (S_6_). 7. HIC-AHP with their weights (S_7_) (Figure 4).

The combination of criteria-maps to obtain the priority map was carried out with the following formula:

(1) $$P_{j}=\sum_{i=1}^{n}W_{i}X_{j}$$

where, $P_{j}$ represents the priority value of cell j, $W_{i}$ represents the weight of criterion i, $X_{j}$ represents the reclassified value of each criterion for cell j, and n represents the total number of criteria.

### 3.6. Identification of Priority Areas and Recommendations

The values of the obtained maps were reclassified on a scale from 0 to 100, an interval used to express the conservation priority (values close to 0 for low priority and close to 100 for high priority). Areas with values between 85 and 100 were considered a priority. This interval was used in a study where a similar methodology was applied in the context of systematic restoration planning [36]. Finally, the areas obtained for each scenario were identified and compared, and recommendations were made that the decision-makers on agrobiodiversity conservation in Mexico can consider.

All rasterization and map editing actions were performed with basic GIS operations (buffering, reclass, rescale, map algebra, etc.) in ArcGIS 10.2.2. [81]. The process for defining priority areas for hawthorn conservation is summarized in five general stages in Figure 4.

## 4. Conclusions

The priority areas for the in situ conservation of *Crataegus* spp. diversity were presented under different scenarios and defined by different sets and sums of criteria. These scenarios coincide in specific areas, and some maintain exclusive areas. The selection of the priority areas depends on the strategy used to determine these areas, the decision-makers, and the resources that may be allocated for this objective.

This study represents the first step in a sequence of actions that may lead to effective and efficient conservation of plant genetic resources of a species of importance for Mexicans. It should be followed by the implementation of in situ conservation projects and programs ideally adapted to the conditions of each site. A methodology was introduced for the design of conservation strategies for hawthorn and other highly important plant genetic resources in Mexico, as a complement to the ex situ conservation carried out in germplasm banks. This type of decision-making instrument will support the construction of public policies for the conservation of agrobiodiversity in Mexico.

## Figures and Tables

**Table 1:** Favorable status, final values, and weight of the important criteria in the identification of priority areas for the in situ conservation of wild hawthorn (*Crataegus* spp.) species, obtained by experts (EXP) and analytic hierarchy process (AHP).

Criterion	Favorable Status	Scale	Weight	
**Biological**			EXP *	AHP *
1. Species richness	High	0, 20, 40, 60, 80, 100	**0.066**	**0.123**
2. Phenotypic diversity	High	0, 20, 40, 60, 80, 100	**0.064**	**0.118**
3. Ecogeographic diversity	High	0, 20, 40, 60, 80, 100	**0.059**	**0.107**
**Socioeconomic**				
4. Percentage of the population lacking access to food	High	0, 25, 50, 75, 100	**0.055**	0.020
5. Percentage of the population living in poverty	High	0, 20, 40, 60, 80, 100	0.035	**0.034**
6. Population density	Low	0, 25, 50, 75, 100	0.029	0.029
7. Level of indigenous presence	High	0, 33, 66, 100	0.039	**0.029**
8. Type of municipality	Rural	0, 100	0.038	**0.092**
9. Percentage of the population employed in agricultural activity	High	0, 25, 50, 75, 100	0.020	0.013
**Ecological**				
10. Protected natural areas (PNAs)	Area not located in PNA	0, 100	**0.058**	**0.100**
11. Level of protection of PNAs	Low level of conservation	0, 25, 50, 75, 100	**0.050**	0.021
12. Terrestrial priority sites for biodiversity conservation	Sites with high priority	0, 33, 66, 100	**0.046**	0.028
13. Eligible areas for biodiversity conservation	Eligible areas	0, 100	0.036	0.011
14. Temperate forest areas	Temperate forest areas	0, 100	0.043	**0.040**
15. Submontane grassland and shrubland areas	Submontane grassland and shrubland areas	0, 100	0.030	**0.029**
**Cultural**				
16. Loss of wild hawthorn ecotypes caused by the lack of knowledge about their uses, lack of consumption, and/or lack of cultivation	Yes, there is a loss specifically due to these causes	0, 100	**0.057**	0.013
17. Number of different types of wild hawthorn	High number	0, 20, 40, 60, 80, 100	**0.056**	**0.099**
18. Loss of different wild hawthorn ecotypes	Yes, there is a loss	0, 100	**0.056**	**0.040**
19. Harvest of wild hawthorn for self-consumption	Harvested for self-consumption	0, 100	**0.046**	0.019
20. Harvest of wild hawthorn for sale	Harvested for sale	0, 100	0.040	0.008
21. Number of uses of wild hawthorn	High number of uses	0, 25, 50, 75, 100	0.045	0.019
22. Number of common names of wild hawthorn	High number of common names	0, 50, 100	0.030	0.007

* Highly important criteria (HIC) are highlighted in bold, which are determined from the threshold obtained by calculating the 50th percentile (0.0455 for experts and 0.0292 for AHP).

**Table 2:** Favorable status, final values, and weight of the important criteria for the identification of priority areas for the in situ conservation of cultivated hawthorn (*C. mexicana*) obtained by experts (EXP) and analytic hierarchy process (AHP).

Criterion	Favorable Status	Scale	Weight	
**Biological**			EXP *	AHP *
1. Phenotypic diversity	High	0, 20, 40, 60, 80, 100	**0.059**	**0.102**
2. Ecogeographic diversity	High	0, 25, 50, 75, 100	**0.056**	**0.104**
3. Species richness	High	0, 20,40, 60, 80, 100	0.034	**0.109**
**Socioeconomic**				
4. Percentage of the population employed in agricultural activity	High	0, 20, 40, 60, 80, 100	0.040	**0.095**
5. Percentage of the population lacking access to food	High	0, 20, 40, 60, 80, 100	0.041	0.017
6. Percentage of elderly people	Low	0, 20, 40, 60, 80, 100	0.033	**0.025**
7. Percentage of the population living in poverty	High	0, 20, 40, 60, 80, 100	0.025	**0.034**
8. Type of municipality	Rural	0, 100	**0.044**	**0.082**
**Ecological**				
9. Protected natural areas (PNAs)	Area not located in PNA	0, 100	0.031	0.015
10. Terrestrial priority sites for biodiversity conservation	Sites with high priority	0, 33, 66, 100	0.030	0.013
11. Rainfed agriculture areas	Rainfed agriculture areas	0, 100	0.037	**0.103**
**Cultural**				
12. Number of varieties of cultivated hawthorn	High number	0, 25, 50, 75, 100	**0.068**	**0.077**
13. Number of uses of cultivated hawthorn	High number	0, 20, 40, 60, 80, 100	**0.057**	0.023
14. Loss of cultivated varieties of hawthorn specifically due to low product prices and change to other fruit trees	There is no loss	0, 100	**0.055**	**0.036**
15. Substitution of landraces by new hawthorn varieties	There is no substitution of landraces	0, 100	**0.054**	0.018
16. Types of cultivated varieties	Mostly traditional varieties (seedling trees)	0, 50, 100	**0.050**	**0.026**
17. Hawthorn cultivation for sale	Not cultivated for sale	0, 100	**0.050**	0.012
18. Number of species associated with hawthorn cultivation	High number	0, 33, 66, 100	**0.046**	**0.035**
19. Seed/plant flow	Yes, there is exchange	0, 100	**0.046**	0.014
20. Association of hawthorn with other crops	Association with other crops	0, 100	**0.044**	0.019
21. Hawthorn cultivation for self-consumption	Cultivated for self-consumption	0, 100	0.043	0.022
22. Estimated age of the established plants	Old age	0, 20, 40, 60, 80, 100	0.032	0.007
23. Number of common names of cultivated hawthorn	High number	0, 100	0.027	0.010

* Highly important criteria (HIC) are highlighted in bold, which are determined from the threshold obtained by calculating the 50th percentile (0.0440 for experts and 0.0249 for AHP).

**Table 3:** Location and surface of priority areas for the conservation of wild hawthorn (*Crataegus* spp.) and cultivated hawthorn (*C. mexicana*) based on seven scenarios. S_1_—All criteria with the same weight. S_2_—All criteria with weights assigned by experts. S_3_—All criteria with weights assigned by AHP. S_4_—HIC-EXP with the same weight. S_5_—HIC-EXP with the weights assigned by experts. S_6_—HIC-AHP with the same weight. S_7_-HIC—AHP with weights assigned by AHP.

Scenario	Area (km^2^)	State/Municipalities
**Wild**		
S_1_	50	Chiapas: San Cristobal de las Casas, Huixtan
S_2_	100	State of Mexico: Xalatlaco, Tianguistenco, Ocuilan, Tochimilco, Atzitzihuacan. Morelos: Tetela del Volcan. Chiapas: Zinacantan
S_3_	100	Chiapas: Zinacantan, Amatenango del Valle, San Cristobal de las Casas
S_4_	50	State of Mexico: Xalatlaco, Tianguistenco, Ocuilan
S_5_	50	State of Mexico: Ocuilan, Tianguistenco, Xalatlaco
S_6_	175	Chiapas: Chamula, Zinacantan, San Cristobal de las Casas, Teopisca, Amatenango del Valle
S_7_	125	State of Mexico: Ocuilan, Tianguistenco. Chiapas: Zinacantan, Chamula, San Cristobal de las Casas, Amatenango del Valle
**Cultivated**		
S_1_	50	Puebla: Chiautzingo, Huejotzingo, Nealtican
S_2_	50	Puebla: Chiautzingo, Huejotzingo, Nealtican
S_3_	150	Puebla: San Felipe Teotlalcingo, Chiautzingo, Huejotzingo, Domingo Arenas, Tlaltenago, Juan C. Bonilla, Calpan, Nealtican, San Nicolas de Los Ranchos, San Salvador El Verde, Acatzingo, General Felipe Alvarez, Soltepec
S_4_	25	Puebla: Calpan, Nealtican, San Nicolas de Los Ranchos
S_5_	75	Puebla: Huejotzingo, Domingo Arenas, Calpan, San Nicolas de Los Ranchos
S_6_	375	Chiapas: Huixtan; Veracruz: Chiconquiaco, Acatlan, Landero and Coss, Calcahualco. Puebla: Acatzingo, Soltepec, Huejotzingo, Domingo Arenas, Calpan, San Nicolas de Los Ranchos, Nealtican
S_7_	325	Chiapas: Hixtan, Tenejapa, San Cristobal de las Casas Puebla: Huejotzingo, Calpan, Domingo Arenas, San Nicolas de Los Ranchos. Veracruz: Calcahualco, Alpatlahuac

S_1_—All criteria with the same weight. S_2_—All criteria with weights assigned by experts. S_3_—All criteria with weights assigned by AHP. S_4_—HIC-EXP with the same weight. S_5_—HIC-EXP with the weights assigned by experts. S_6_—HIC-AHP with the same weight. S_7_—HIC-AHP with weights assigned by AHP.

## Data Availability

Data is contained within the article and Supplementary Material.

## Supplementary Materials

The following are available online at https://www.mdpi.com/article/10.3390/plants10122561/s1, Table S1. Description and rasterization method of the criteria subjected to the selection and prioritization process to identify priority areas for the conservation of *Crataegus* spp.
