# Supplementary material for: Multi-Criteria Analysis for the Prioritization of Areas for the In Situ Conservation of Crataegus L., an Underutilized Fruit Tree in Mexico
Source: Plants (Basel). 2021 Nov 23;10(12):2561. doi: 10.3390/plants10122561 (PMC8706608; doi:10.3390/plants10122561)
Supplement: Supplementary file 1 [file plants-10-02561-s001.zip › plants-1460014-supplementary.pdf]

Table S1. Description and rasterization method of the criteria subjected to the selection and prioritization process to identify priority areas for the conservation of *Crataegus* spp.

Criterion	Description	Source	Original layer format/ Rastering method
**Biological**			
Species richness	Number of different species that occur in a specific area.	Created with DIVA-GIS 7.0 [82]	Raster/NA
Phenotypic diversity	Variation level of morphological characteristics that occur in a specific area.	[51] Created with DIVmaps [83]	Raster/NA
Ecogeographic diversity	Variation level of contrasting environmental conditions (different adaptations) per unit area.	Created with DIVmaps [83]	Raster/NA
**Socioeconomic**			
Percentage of the population lacking access to food	(Number of people lacking access to food/Total population of the municipality) *100	[69]	Vectorial (Polygon)/ MAXIMUM AREA
Percentage of the population living in poverty	(Number of people in poverty/Total population of the municipality) *100	[70]	Vectorial (Polygon)/ MAXIMUM AREA
Level of social exclusion	Level of deficiencies suffered by the population caused by the lack of access to education, the residence in inadequate housings, insufficient monetary income perception, and the related to the residence in small towns.	[70]	Vectorial (Polygon)/ MAXIMUM AREA
Level of social backwardness	Relationship of four variables per municipality [1) educational lag; 2) access to health services; 3) access to basic, quality services and spaces in the home, and 4) assets in the home].	[70]	Vectorial (Polygon)/ MAXIMUM AREA
Male and female population ratio	(Male population at the municipal level/female population at the municipal level).	[71]	Vectorial (Polygon)/ MAXIMUM AREA
Percentage of elderly people	(People within an age range from 40 to 64 years at the municipal level/total population of the municipality) *100	[71]	Vectorial (Polygon)/ MAXIMUM AREA
Population density	Indicates the number of people per unit area.	[72]	Vectorial (Polygon)/MAXIMUM AREA
Level of indigenous presence	Proportion of indigenous population per municipality.	[73]	Vectorial (Polygon)/ MAXIMUM AREA
Human development index value	Level of capabilities and freedom to have an acceptable quality of life.	[74]	Vectorial (Polygon)/ MAXIMUM AREA
Percentage of cumulative migration	(People who were born in another geographic entity and who reside in the municipality/total population of the municipality) *100	[72]	Vectorial (Polygon)/ MAXIMUM AREA
Type of municipality	It represents the type of municipality that derives from the relationship between the number of inhabitants per unit area. It can be a rural or urban municipality.	[75]	Vectorial (Polygon)/ MAXIMUM AREA

Percentage of the population employed in agricultural activity	(Population employed in agricultural activity at the municipal level/total employed population at the municipal level) *100	[76]	Vectorial (Polygon)/ MAXIMUM AREA
**Ecological**			
Protected natural areas (PNAs)	Established areas to protect national biodiversity.	[20]	Vectorial (Polygon)/ MAXIMUM AREA
Level of protection of PNAs	Level of protection that a protected area exercises over the natural resources that occur in it.	[20]	Vectorial (Polygon)/ MAXIMUM AREA
Terrestrial priority sites for biodiversity conservation	Areas that, according to their biodiversity indices, represent an extreme or low priority for their conservation.	[21]	Vectorial (Polygon)/ MAXIMUM AREA
Eligible areas for biodiversity conservation	Areas with payments for environmental services to protect biodiversity.	[77]	Vectorial (Polygon)/ MAXIMUM AREA
Rainfed agriculture areas	Areas where agriculture is rainfed because of the availability of rainwater during the agricultural cycle.	[78]	Vectorial (Polygon)/ MAXIMUM AREA
Temperate forest areas	Areas with plant communities dominated by tall trees, mostly pine and oak, accompanied by several other species that inhabit mountainous areas with a temperate to cold climate.	[78]	Vectorial (Polygon)/ MAXIMUM AREA
Submontane grassland and shrubland areas	Areas with plant communities that are a product of forest land clearing.	[78]	Vectorial (Polygon)/ MAXIMUM AREA
**Cultural for wild hawthorn**			
Common names of wild hawthorn	Number of common names assigned to hawthorn in a specific area.	Surveys	Vectorial (Points)/ MAXIMUM
Different wild hawthorn ecotypes	Number of different types of wild hawthorn that the farmer identifies in specific areas.	Surveys	Vectorial (Points)/ MAXIMUM
Loss of different wild hawthorn ecotypes	According to the farmer's perception, there is or is not a decrease in the types of wild hawthorn that he recognizes in specific areas.	Surveys	Vectorial (Points)/ MOST FREQUENT
Loss of wild hawthorn ecotypes specifically caused by the lack of knowledge about their uses, lack of consumption, and/or lack of cultivation	The specific cause of the loss of wild ecotypes of hawthorn is the lack of knowledge about its uses, consumption, and cultivation in specific areas.	Surveys	Vectorial (Points)/ MOST FREQUENT
Harvest of wild hawthorn for self-consumption	The main reason why wild hawthorn is harvested in specific areas is for self-consumption.	Surveys	Vectorial (Points)/ MOST FREQU
Harvest of wild hawthorn for sale	The main reason why wild hawthorn is harvested in specific areas is for sale.	Surveys	Vectorial (Points)/ MOST FREQUENT
Uses of wild hawthorn	Number of different known uses of wild hawthorn in a specific area (it does not necessarily refer to its uses there).	Surveys	Vectorial (Points)/ MAXIMUM
**Cultural for cultivated hawthorn**			
Varieties of cultivated hawthorn	Number of cultivated varieties of hawthorn in specific areas.	Surveys	Vectorial (Points)/MAXIMUM

Loss of cultivated varieties of hawthorn	According to the farmer's perception, there is or is not a decrease in the cultivated varieties of hawthorn that he recognizes in specific areas.	Surveys	Vectorial (Points)/ MOST FREQUENT
Loss of cultivated varieties of hawthorn specifically due to low product prices and change to other fruit trees	Loss of cultivated varieties of hawthorn specifically due to low product prices and change to other fruit trees.	Surveys	Vectorial (Points)/ MOST FREQUENT
Substitution of landraces by new hawthorn varieties	There is a substitution of native varieties for new varieties of hawthorn in specific areas.	Surveys	Vectorial (Points)/ MOST FREQUENT
Hawthorn cultivation for self-consumption	The main reason why hawthorn is grown in specific areas is for self-consumption.	Surveys	Vectorial (Points)/ MOST FREQUENT
Hawthorn cultivation for sale	The main reason why hawthorn is grown in specific areas is for sale.	Surveys	Vectorial (Points)/ MOST FREQUENT
Uses of cultivated hawthorn	Number of different known uses of cultivated hawthorn in a specific area (it does not necessarily refer to the uses that are given there).	Surveys	Vectorial (Points)/ MAXIMUM
Number of established plants	Number of established hawthorn plants per productive unit.	Surveys	Vectorial (Points)/ MEAN
Estimated age of the established plants	Estimated age of the established plants per productive unit.	Surveys	Vectorial (Points)/ MEAN
Association of hawthorn with other crops	Crops of only hawthorn or associated with other fruit trees in productive units.	Surveys	Vectorial (Points)/ MOST FREQUENT
Species associated with hawthorn cultivation	Number of species associated with hawthorn cultivation in productive units.	Surveys	Vectorial (Points)/ MAXIMUM
Types of cultivated varieties	Type of hawthorn varieties grown in specific areas according to their origin, they can be traditional (seedling trees) or selected.	Surveys	Vectorial (Points)/ MOST FREQUENT
Common names of cultivated hawthorn	Number of common names assigned to hawthorn in a specific area.	Surveys	Vectorial (Points)/ MAXIMUM
Seed/Plant flow	There is or there is not an exchange of hawthorn seeds or plants among producers.	Surveys	Vectorial (Points)/MAXIMUM

References

1. Toledo, Á.; Burlingame, B. Biodiversity and nutrition: A common path toward global food security and sustainable development. *J. Food Compost. Anal.* **2006**, *19*, 477–483. https://doi.org/10.1016/j.jfca.2006.05.001.
2. Thrupp, L.A. Linking agricultural biodiversity and food security: The valuable role of agrobiodiversity for sustainable agriculture. *Int. Aff.* **2000**, *76*, 265–281. https://doi.org/10.1111/1468-2346.00133.
3. Lobo, M.; Medina, C.I. Conservación de recursos genéticos de la agrobiodiversidad como apoyo al desarrollo de sistemas de producción sostenibles. *Cienc. Tecnol. Agropecu.* **2009**, *10*, 33–42. https://doi.org/10.21930/rcta.vol10_num1_art:126.
4. de Carvalho, M.Â.; Bebeli, P.J.; da Silva, A.M.; Bettencourt, E.; Slaski, J.J.; Dias, S. Agrobiodiversity: The importance of inventories in the assessment of crop diversity and its time and spatial changes. In *Genetic Diversity and Erosion in Plants*; Ahuja, M.R., Jain, S.M., Eds.; Springer: Cham, Switzerland, 2016; Volume 8, pp. 307–335. https://doi.org/10.1007/978-3-319-25954-3_9.

5. Bellon, M.R.; Barrientos-Priego, A.F.; Colunga-García-Marín, P.; Perales, H.; Reyes Agüero, J.A.; Rosales-Serna, R.; Zizumbo-Villarreal, D. Diversidad y conservación de recursos genéticos en plantas cultivadas. In *Capital Natural de Mexico: Estado de Conservación y Tendencias de Cambio*; CONABIO, Ed.; CONABIO: Mexico City, Mexico, 2009; Volume 2, pp. 355–382.
6. Koleff, P.; Tambutti, M.; March, I.J.; Esquivel, R.; Cantú, C.; Lira-Noriega, A. Identificación de prioridades y análisis de vacíos y omisiones en la conservación de la biodiversidad de Mexico. In *Capital Natural de Mexico: Estado de Conservación y Tendencias de Cambio*; CONABIO, Ed.; CONABIO: Mexico City, Mexico, 2009; Volume 2, pp. 651–718.
7. Maxted, N.; Guarino, L.; Myer, L.; Chiwona, E.A. Towards a methodology for on-farm conservation of plant genetic resources. *Genet. Resour. Crop. Evol.* **2002**, *49*, 31–46. https://doi.org/10.1023/A:1013896401710.
8. FAO. *Second Report on the State of the World's Plant Genetic Resources for Food and Agriculture*; Food and Agriculture Organization of the United Nations: Rome, Italy, 2010; p. 372. Available online: http://www.fao.org/docrep/014/i1500s/i1500s.pdf (accessed on 26 February 2018).
9. Maxted, N.; Ford-Lloyd, B.V.; Hawkes, J.G. (Eds.) Complementary conservation strategies. In *Plant Genetic Conservation: The In-Situ Approach*; Chapman & Hall: London, UK, 1997; pp. 15–40. https://doi.org/10.1007/978-94-009-1437-7.
10. Geneletti, D.; Orsi, F.; Lanni, E.; Newton, A.C. Identificación de áreas prioritarias para la restauración de bosques secos. In *Principios y Práctica de la Restauración del Paisaje Forestal: Estudios de Caso en las Zonas Secas de América Latina*; Newton, A.C., Tejedor, N., Eds.; UICN, Fundación Internacional Para la Restauración de Ecosistemas: Gland, Switzerland, 2011; pp. 289–326.
11. Chávez, H.; González, M.J.; Hernández de la Rosa, P. Metodologías para identificar áreas prioritarias para conservación de ecosistemas naturales. *Rev. Mex. Cienc. For.* **2015**, *6*, 8–23. https://doi.org/10.29298/rmcf.v6i27.277.
12. Ceballos, G.; Díaz-Pardo, E.; Espinosa, H.; Flores-Villela, O.; García. A.; Martínez, L. Zonas críticas y de alto riesgo para la conservación de la biodiversidad de Mexico. In *Capital Natural de Mexico: Estado de Conservación y Tendencias de Cambio*, CONABIO, Ed.; CONABIO: Mexico City, Mexico, 2009; Volume 2, pp. 575–600.
13. Arriaga-Cabrera, L.; Aguilar, V.; Espinoza, J.M.; Galindo, C.; Herrmann, H.; Santana, E. Regiones prioritarias y planeación para la conservación de la biodiversidad. In *Capital Natural de Mexico: Estado de Conservación y Tendencias de Cambio*; CONABIO, Ed.; CONABIO: Mexico City, Mexico, 2009; Volume 2, pp. 433–57.
14. Regan, H.M.; Davis, F.W.; Andelman, S.J.; Widyanata, A.; Freese, M. Comprehensive criteria for biodiversity evaluation in conservation planning. *Biodivers Conserv.* **2007**, *16*, 2715–2728. https://doi.org/10.1007/s10531-006-9100-3.
15. Geneletti, D. A GIS-based decision support system to identify nature conservation priorities in an alpine valley. *Land Use Policy* **2004**, *21*, 149–160. https://doi.org/10.1016/j.landusepol.2003.09.005.
16. Moffett, A.; Sarkar, S. Incorporating multiple criteria into the design of conservation area networks: A minireview with recommendations. *Divers Distrib.* **2006**, *12*, 125–137. https://doi.org/10.1111/j.1366-9516.2005.00202.x.
17. Bottero, M.; Comino, E.; Duriavig, M.; Ferretti, V.; Pomarico, S. The application of a Multicriteria Spatial Decision Support System (MCSDSS) for the assessment of biodiversity conservation in the Province of Varese (Italy). *Land Use Policy* **2013**, *30*, 730–738. https://doi.org/10.1016/j.landusepol.2012.05.015.
18. Koleff, P.; Urquiza, T. *Planeación Para la Conservación de la Biodiversidad Terrestre en Mexico: Retos en un País Megadiverso*; CONABIO-CONANP: Ciudad de Mexico, Mexico, 2011; p. 244.
19. March, I.J.; Carvajal, M.A.; Vidal, R.M.; San Román, J.E.; Ruiz, G. Planificación y desarrollo de estrategias para la conservación de la biodiversidad. In *Capital Natural de Mexico: Estado de Conservación y Tendencias de Cambio*; CONABIO, Ed.; CONABIO: Mexico City, Mexico, 2009; Volume 2, pp. 545–573.
20. CONANP. Listado de las Áreas Naturales Protegidas de Mexico (LISTANP). Available online: http://sig.conanp.gob.mx/website/pagsig/listanp/ (accessed on 29 March 2018).
21. CONABIO. Sitios de Atención Prioritaria Para la Conservación de la Biodiversidad, Escala 1:1000,000. Available online: http://www.conabio.gob.mx/informacion/metadata/gis/sap_gw.xml?_htt-cache=yes&_xsl=/db/metadata/xsl/fgdc_html.xsl&_indent=no (accessed on 23 January 2018).
22. CONANP. *Programa de Conservación de Maíz Criollo en MEXICO*; SEMARNAT: Ciudad de Mexico, Mexico, 2016; p. 31. Available online: https://www.conanp.gob.mx/maiz_criollo/maiz%20final.pdf (accessed on 21 February 2018).
23. Contreras-Toledo, A.R.; Cortés-Cruz, M.; Costich, D.E.; de Lourdes Rico-Arce, M.; Brehm, J.M.; Maxted, N. Diversity and conservation priorities of crop wild relatives in Mexico. *Plant Genet. Resour.* **2019**, *17*, 140–150. https://doi.org/10.1017/S1479262118000540.
24. Santos, J.M. El planteamiento teórico multiobjetivo/multicriterio y su aplicación a la resolución de problemas medioambientales y territoriales, mediante los SIG raster. *Ser. VI Geogr.* **1997**, *10*, 129–151. https://doi.org/10.5944/etfvi.10.1997.2547.
25. Beinat, E. Multi-criteria analysis for environmental management. *J. Multi-Criteria Decis. Anal.* **2001**, *10*, 51. https://doi.org/10.1002/mcda.294.
26. Dykstra, D.P. *Mathematical Programming for Natural Resource Management*; McGraw-Hill Book Co.: New York, NY, USA, 1984; Volume 7, p. 318.
27. Locatelli, B.; Rojas, V.; Salinas, Z. Impacts of payments for environmental services on local development in northern Costa Rica: A fuzzy multi-criteria analysis. *For. Policy Econ.* **2008**, *10*, 275–285. https://doi.org/10.1016/j.forpol.2007.11.007.

28. Geneletti, D. An approach based on spatial multicriteria analysis to map the nature conservation value of agricultural land. *J. Environ. Manag.* **2007**, *83*, 228–235. https://doi.org/10.1016/j.jenvman.2006.03.002.
29. Brown. K.; Adger, W.N.; Tompkins, E.; Bacon, P.; Shim, D.; Young, K. Trade-off analysis for marine protected area management. *Ecol. Econ.* **2001**, *37*, 417–434. https://doi.org/10.1016/S0921-8009(00)00293-7.
30. Villa, F.; Tunesi, L.; Agardy, T. Zoning marine protected areas through spatial multiple-criteria analysis: The case of the Asinara Island national marine reserve of Italy. *Conserv. Biol.* **2002**, *16*, 515–526. https://doi.org/10.1046/j.1523-1739.2002.00425.x.
31. Wood, L.J.; Dragicevic, S. GIS-based multicriteria evaluation and fuzzy sets to identify priority sites for marine protection. *Biodivers Conserv.* **2007**, *16*, 2539–2558. https://doi.org/10.1007/s10531-006-9035-8.
32. Castellini, C.; Boggia, A.; Cortina, C.; Dal Bosco, A.; Paolotti, L.; Novelli, E. A multicriteria approach for measuring the sustainability of different poultry production systems. *J. Clean. Prod.* **2012**, *37*, 192–201. https://doi.org/10.1016/j.jclepro.2012.07.006.
33. van der Horst, D.; Gimona, A. Where new farm woodlands support biodiversity action plans: A spatial multi-criteria analysis. *Biol. Conserv.* **2005**, *123*, 421–432. https://doi.org/10.1016/j.biocon.2004.11.020.
34. Sarkar, S.; Pressey, R.L.; Faith, D.P.; Margules, C.R.; Fuller, T.; Stoms, D.M. Biodiversity conservation planning tools: Present status and challenges for the future. *Annu. Rev. Environ. Resour.* **2006**, *31*, 123–159. https://doi.org/10.1146/annurev.energy.31.042606.085844.
35. Tapia Bastidas, C.G. Identificación de Áreas Prioritarias Para la Conservación de Razas de Maíz en la Sierra de Ecuador. Ph.D. Thesis, Universidad Politécnica de Madrid, Madrid, Spain, 2015.
36. Tobón, W.; Koleff, P.; Urquiza-Haas, T.; Méndez, G.G. Propuesta metodológica para identificar prioridades de restauración en Mexico. In *Experiencias Mexicanas en la Restauración de los Ecosistemas*; Ceccon, E., Martínez-Garza, C., Eds.; CONABIO: Ciudad de Mexico, Mexico, 2016, pp. 31–48.
37. Urquiza-Haas, T.; Kolb, M.; Koleff, P.; Lira-Noriega, A.; Alarcón, J. Methodological approach to identify Mexico's terrestrial priority sites for conservation. *Gap Anal. Program Brief* **2009**, *16*, 60–70.
38. Núñez-Colín, C.A. Áreas prioritarias para colectar germoplasma de (*Crataegus* L.) en Mexico con base en la diversidad y riqueza de especies. *Agric. Técnica Méx.* **2009**, *35*, 333–338.
39. Phipps, J.B.; O'Kennon, R.J.; Lance, R.W. *Hawthorns and Medlars*; Royal Horticultural Society: Cambridge, UK, 2003.
40. Phipps, J.B. *Monograph of Northern Mexican Crataegus (Rosaceae Subfam. Maloideae*; Sida Botanical Miscellany 15, Botanical Research Institute of Texas: Fort Worth, TX, USA, 1997.
41. Núñez-Colín, C.A.; Hernández-Martínez, M.Á. La problemática en la taxonomía de los recursos genéticos de tejocote (*Crataegus* spp.) en Mexico. *Rev. Mex. Cienc. Agríc.* **2011**, *2*, 141–153.
42. Cabrera, L. *Diccionario de Aztequismos*; Colofón: Ciudad de Mexico, Mexico, 1992.
43. Núñez-Colín, C.A.; Nieto-Ángel, R.; Barrientos-Priego, A.F.; Segura, S.; Sahagún-Castellanos, J.; González-Andrés, F. Distribución y caracterización eco-climática del género *Crataegus* L. (Rosaceae, Subfam. Maloideae) en Mexico. *Rev. Chapingo Ser. Hortic.* **2008**, *14*, 177–184. https://doi.org/10.5154/r.rchsh.2006.06.027.
44. Banderas-Tarabay, A.J.; Cervantes-Rodríguez, M.; Méndez-Iturbide, D. Biological properties and antioxidant activity of hawthorn *Crataegus mexicana*. *J. Pharm. Pharm.* **2015**, *6*, 1–8. http://dx.doi.org/10.4172/2153-0645.1000153.
45. Kumar, D.; Arya, V.; Bhat, Z.A.; Khan, N.A.; Prasad, D.N. The genus *Crataegus*: Chemical and pharmacological perspectives. *Rev. Bras. Farm.* **2012**, *22*, 1187–1200. https://doi.org/10.1590/S0102-695X2012005000094.
46. Nuñez-Colín, C.A.; Sánchez Vidaña, D.I. Ethnobotanical, cultural, and agricultural uses of tejocote (*Crataegus* species) in Mexico. XXVIII International Horticultural Congress on Sciences and Horticulture for people: III International Symposium on Plant Genetic Resources. *Acta Hortic.* **2011**, 918. https://doi.org/10.17660/ActaHortic.2011.918.118.
47. Argeta, V.A.; Cano, A.L. *Atlas de las Plantas de la Medicina Tradicional Mexicana*; Instituto Nacional Indigenista: Mexico City, Mexico, 1994.
48. Nieto-Ángel, R. (Ed.) Colección, conservación y caracterización del tejocote (*Crataegus* spp.). In *Frutales Nativos, un Recurso Fitogenético de Mexico*; Universidad Autónoma Chapingo: Estado de Mexico, Mexico, 2007; pp. 25–107.
49. SIAP. Sistema de Información Agroalimentaria y Pesquera. Available online: http://www.siap.gob.mx/cierre-de-la-produccion-agricola-por-cultivo/ (accessed on 29 January 2018).
50. SNICS. *Catálogo Nacional de Variedades Vegetales 29*; SNICS-SAGARPA: Estado de Mexico, Mexico, 2012.
51. Betancourt-Olvera, M.; Nieto-Ángel, R.; Urbano, B.; González-Andrés, F. Analysis of the biodiversity of hawthorn (*Crataegus* spp.) from the morphological, molecular, and ethnobotanical approaches, and implications for genetic resource conservation in scenery of increasing cultivation: The case of Mexico. *Genet. Resour. Crop. Evol*. **2018**, *65*, 897–916. https://doi.org/10.1007/s10722-017-0583-4.
52. IUCN. The IUCN Red List of Threatened Species. Version 2021-1. Available online: https://www.iucnredlist.org (accessed on 14 August 2021).

53. Bonn, A.; Gaston, K.J. Capturing biodiversity: Selecting priority areas for conservation using different criteria. *Biodivers Conserv.* **2005**, *14*, 1083–1100. https://doi.org/10.1007/s10531-004-8410-6.
54. Margules, C.R.; Pressey, R.L.; Williams, P.H. Representing biodiversity: Data and procedures for identifying priority areas for conservation. *J. Biosci.* **2002**, *27*, 309–326. https://doi.org/10.1007/BF02704962.
55. Asaad, I.; Lundquist, C.J.; Erdmann, M.V.; Costello, M.J. Ecological criteria to identify areas for biodiversity conservation. *Biol. Conserv.* **2017**, *213*, 309–316. https://doi.org/10.1016/j.biocon.2016.10.007.
56. Pacicco, L.; Bodesmo, M.; Torricelli, R.; Negri, V. A methodological approach to identify agro-biodiversity hotspots for priority in situ conservation of plant genetic resources. *PLoS ONE* **2018**, *13*, 1–20. https://doi.org/10.1371/journal.pone.0197709.
57. Bonneuil, C.; Goffaux, R.; Bonnin, I.; Montalent, P.; Hamon, C.; Balfourier, F. A new integrative indicator to assess crop genetic diversity. *Ecol. Indic.* **2012**, *23*, 280–289. https://doi.org/10.1016/j.ecolind.2012.04.002.
58. Adams, W.M.; Aveling, R.; Brockington, D.; Dickson, B.; Elliott, J.; Hutton, J. Biodiversity conservation and the eradication of poverty. *Science* **2004**, *306*, 1146–1149. https://doi.org/10.1126/science.1097920.
59. Ding, Y. Impacts of Affluence and Overexploitation of Natural Resources. Environment and Development. In *Encyclopedia of Life Support Systems*; UNESCO: Paris, France, 2003.
60. López, G.; Palomino, B. Políticas públicas y ecoturismo en comunidades indígenas de Mexico. *Teoría Y Prax.* **2008**, *5*, 33–50. https://doi.org/10.22403/UQROOMX/TYP05/03.
61. Ramírez-Galindo, J.; Cruz-Castillo, J.G.; Gallegos-Vázquez, C.; Espíndola-Barquera, M.d.l.C.; Nieto-Ángel, R.; Avendaño-Arrazate, C.H. *Conservación y Aprovechamiento Sostenible de Frutales Nativos de Mexico*, SNICS Y UACh: Estado de Mexico, Mexico, 2016; pp. 156.
62. FAO. *Second Global Plan of Action for Plant Genetic Resources for Food and Agriculture*; FAO: Rome, Italy, 2011; p. 322. Available online: http://www.fao.org/docrep/015/i2624e/i2624e00.pdf (accessed on 11 April 2018).
63. Kell, S.P.; Maxted, N.; Frese, L.; Iriondo, J.M. In situ conservation of crop wild relatives: A strategy for identifying priority genetic reserve sites. In *Agrobiodiversity Conservation: Securing the Diversity of Crop Wild Relatives and Landraces*; Maxted, N., Dulloo, M.E., Ford-Lloyd, B.V., Frese, L., Iriondo, J.M., Pinheiro de Carvalho, M.A.A., Eds.; CABI: Wallingford, UK, 2012; pp. 7–19. https://doi.org/10.1079/9781845938512.0007.
64. Brehm, J.M.; Maxted, N.; Martins-Loução, M.A.; Ford-Lloyd, B.V. New approaches for establishing conservation priorities for socio-economically important plant species. *Biodivers Conserv.* **2010**, *19*, 2715–2740. https://doi.org/10.1007/s10531-010-9871-4.
65. Maxted, N.; Ford-Lloyd, B.V.; Jury, S.; Kell, S.; Scholten, M. Towards a definition of a crop wild relative. *Biodivers Conserv.* **2006**, *15*, 2673–2685. https://doi.org/10.1007/s10531-005-5409-6.
66. Contreras-Toledo, A.R.; Cortés-Cruz, M.A.; Costich, D.; Rico-Arce, M.L.; Brehm, J.M.; Maxted, N. A crop wild relative inventory for Mexico. *Crop. Sci.* **2018**, *58*, 1292–1305. https://doi.org/10.2135/cropsci2017.07.0452.
67. Kremer, I.; Mansour, Y.; Perry, M. Implementing the "wisdom of the crowd". *J. Polit. Econ.* **2014**, *122*, 988–1012. https://doi.org/10.1086/676597.
68. GBIF.org. GBIF Occurrence Download. Available online: https://www.gbif.org/occurrence/download/0004511-160526112335914 (accessed on 08 January 2016).
69. CONABIO. Población con Carencia por Acceso a la Alimentación por Municipio 2010, escala: 1:250,000. Available online: http://www.conabio.gob.mx/informacion/metadata/gis/acalim10gw.xml?_httpcache=yes&_xsl=/db/metadata/xsl/fgdc_html.xsl&_indent=no (accessed on 23 January 2018).
70. CONABIO. Población en Condición de Pobreza por Municipio 2010, Escala: 1:250,000. Available online: http://www.conabio.gob.mx/informacion/metadata/gis/pobzmun10gw.xml?_httpcache=yes&_xsl=/db/metadata/xsl/fgdc_html.xsl&_indent=no (accessed on 23 January 2018).
71. CONABIO. Características Sociodemográficas de Mexico por Municipio, 2010, Escala: 1:250,000. Available online: http://www.conabio.gob.mx/informacion/metadata/gis/pobindigw.xml?_httpcache=yes&_xsl=/db/metadata/xsl/fgdc_html.xsl&_indent=no (accessed on 23 January 2018).
72. CONABIO. Distribución de la Población en Mexico por Municipio 2010, Escala: 1:250,000. Available online: http://www.conabio.gob.mx/informacion/metadata/gis/dipomun10gw.xml?_httpcache=yes&_xsl=/db/metadata/xsl/fgdc_html.xsl&_indent=no (accessed on 23 January 2018).
73. CONABIO. Presencia de la Población Indígena por Municipio 2010, Escala: 1:250,000. Available online: http://www.conabio.gob.mx/informacion/metadata/gis/presindigw.xml?_httpcache=yes&_xsl=/db/metadata/xsl/fgdc_html.xsl&_indent=no (accessed on 23 January 2018).
74. CONABIO. Índice de Desarrollo Humano Por Municipio 2010, Escala: 1:250,000. Available online: http://www.conabio.gob.mx/informacion/metadata/gis/idhmun10gw.xml?_httpcache=yes&_xsl=/db/metadata/xsl/fgdc_html.xsl&_indent=no (accessed on 23 January 2018).
75. CONABIO. Tipología Municipal Por Asentamiento Humano 2014, Escala: 1:250,000. Available online: http://www.conabio.gob.mx/informacion/metadata/gis/tipmun14gw.xml?_httpcache=yes&_xsl=/db/metadata/xsl/fgdc_html.xsl&_indent=no (accessed on 23 January 2018).
76. CONABIO. Actividades Económicas en Mexico Por Municipio 2010, Escala: 1:250,000. Available online: http://www.conabio.gob.mx/informacion/metadata/gis/actmun10gw.xml?_httpcache=yes&_xsl=/db/metadata/xsl/fgdc_html.xsl&_indent=no (accessed on 23 January 2018).

77. CONABIO. Áreas Elegibles Para la Conservación en Mexico 2014, Escala: 1:250,000. Available online: http://www.conabio.gob.mx/informacion/metadata/gis/aecon14gw.xml?_httpcache=yes&_xsl=/db/metadata/xsl/fgdc_html.xsl&_indent=no (accessed on 23 January 2018).
78. INEGI. Uso del Suelo y Vegetación, Escala 1:250,000, Serie IV. Available online: http://www.conabio.gob.mx/informacion/metadata/gis/usv250s6gw.xml?_httpcache=yes&_xsl=/db/metadata/xsl/fgdc_html.xsl&_indent=no (accessed on 27 January 2018).
79. Sipahi, S.; Timor, M. The analytic hierarchy process and analytic network process: An overview of applications. *Manag. Decis.* **2010**, *48*, 775–808. https://doi.org/10.1108/00251741011043920.
80. Saaty, T.L. Decision making—the Analytic Hierarchy and Network Processes (AHP/ANP). *J. Syst. Sci. Syst. Eng.* **2004**, *13*, 1–35. https://doi.org/10.1007/s11518-006-0151-5.
81. ESRI. *ArcGIS Desktop: Release 10.2.2 Redlands*; Environmental Systems Research Institute: West Redlands, CA, USA, 2014.
82. Scheldeman, X.; van Zonneveld, M. *Training Manual on Spatial Analysis of Plant Diversity and Distribution*; Bioversity International: Rome, Italy, 2011; p. 79.
83. Parra-Quijano, M.; Torres, E.; Iriondo, J.M.; López, F.; Molina, A. *CAPFITOGEN Tools. User Manual Version 2.0*; FAO: Rome, Italy, 2015; p. 251.
